# Sociopolitical Development among Latinx Child Farmworkers

**DOI:** 10.3390/youth4020037

**Published:** 2024-04-18

**Authors:** Parissa J. Ballard, Stephanie S. Daniel, Taylor J. Arnold, Jennifer W. Talton, Joanne C. Sandberg, Sara A. Quandt, Melinda F. Wiggins, Camila A. Pulgar, Thomas A. Arcury

**Affiliations:** 1 Department of Family and Community Medicine, Wake Forest University School of Medicine, Winston-Salem, NC 27157, USA;; 2 Department of Biostatistics and Data Science, Division of Public Health Sciences, Wake Forest University School of Medicine, Winston-Salem, NC 27157, USA;; 3 Department of Epidemiology and Prevention, Division of Public Health Sciences, Wake Forest University School of Medicine, Winston-Salem, NC 27157, USA;; 4 Student Action with Farmworkers, 1200 Spaulding Street, Durham, NC 27701, USA;

**Keywords:** sociopolitical development, youth, Latinx child farmworkers, civic attitudes, civic behavior

## Abstract

The objectives of the present study were to describe civic attitudes and behaviors among Latinx child farmworkers in North Carolina, examine civic outcomes across relevant demographic characteristics, and discuss the implications for research on sociopolitical development among Latinx child farmworkers and for developmental theory. Descriptive statistics (count, percent, or mean, standard deviation as appropriate) were calculated for demographic and civic variables. Associations between the demographic variables and the four civic summary variables were calculated using Generalized Linear Models, the Kruskal–Wallis test, t-tests, or Chi-Square tests. Latinx child farmworkers in North Carolina (*N* = 169; ages 11–19, *M*_*a*ge_ = 15.8, 62.7% boys) endorsed relatively high levels of beliefs that society is fair and connections/efficacy in their communities. They reported relatively low involvement in volunteering and political activity. Future work should examine how the daily lives and experiences of child farmworkers inform their developing ideas about civic life in the US and their behavioral participation as they mature.

Sociopolitical development is linked with health and well-being, especially among populations marginalized by systems and structures [1–3], and may be a tool for promoting both individual and community health and the pursuit of health equity [4,5]. Sociopolitical development refers to the processes primarily during adolescence and adulthood whereby people, especially those from historically marginalized groups, gain understanding of cultural and political systems that shape their status in society and eventually take action to transform and reclaim power in those systems [6,7]. Foundational to this process for adolescents is developing attitudes and beliefs about civic life, and taking part in civic behaviors [8,9]. Young people are offered drastically different opportunities to participate in civic life, and, in turn, form different attitudes and beliefs based on experiences in their specific contexts. Understanding how sociopolitical development happens in context, and how it relates to other domains of development, is central in research on youth sociopolitical development and furthers the goal of supporting equitable access and participation in civic life [9]. This study presents descriptive data about civic attitudes and behaviors among Latinx child farmworkers in North Carolina, a unique population that experiences many health inequities [10]. These children have received little empirical attention in sociopolitical development research. As they are an understudied population with regards to sociopolitical development, documenting civic attitudes and behaviors among this population can form a foundation for further research investigating how sociopolitical development occurs in the context of farm work, how it is associated with other domains of healthy development for child farmworkers, and how sociopolitical development may provide child farmworkers with the civic skills to advocate against health inequities they may face.

## 1. The Importance of Sociopolitical Development

The sociopolitical development of youth, which can support active civic participation, is vital to society’s functioning, to individual healthy development, and to equitable opportunities to exercise social and political power. Two important indicators of sociopolitical development are what young people believe and how they feel about society (civic attitudes) and how they participate in their communities (civic behaviors). Civic attitudes include beliefs about societal fairness and inequality, feelings of connection to community, and a sense of efficacy in working for the betterment of the community. Civic attitudes such as feeling connected to one′scommunity and feeling that one can make changes may predict more engaged civic behavior, and more awareness of societal inequality (i.e., the belief that society is not fair) has been found to predict civic behaviors aimed at changing or challenging systems [11–14]. Examples of civic behaviors available to young people include political activities and volunteering. Importantly, volunteering activities are often apolitical and thus differ from political activities in many ways [15]. Having the opportunity to form attitudes and to interact with community shape individual and community development and predict further sociopolitical development as youth age into formal political participation [4,16]. Civic attitudes and behavior are reciprocally related where attitudes—both favorable and unfavorable towards society—can motivate behavior, and behavior informs the subsequent development of attitudes [17]. At a given point in time, the association between attitudes and behavior provides a snapshot of how these aspects of sociopolitical development are forming.

Civic attitudes and behaviors are important developmental outcomes to understand in their own right as they indicate investment in communities. Civic behaviors shape and sustain community and democratic functioning and collectively contribute to changing policies that affect young people and their families. In addition, civic attitudes and behaviors are related to other important developmental domains such as health, well-being, and educational outcomes in nuanced ways depending on the specific identities, contexts, and experiences young people have. For example, young people who participate in civic action in the form of volunteering have more positive subsequent well-being and educational trajectories, while those involved in critical forms of civic action, such as activism, experience both empowerment and educational benefits as well as stress and challenges to well-being (e.g., [3,18–21]). Importantly, sociopolitical development is critical for the health of society as well as for healthy individual development. Young people empowered for civic participation can take collective action to implement or change policies and work for the betterment of their communities.

## 2. Sociopolitical Development Happens in Developmental Micro-Contexts

Based in ecological and sociocultural perspectives of human development [1,22–24], the experiences that young people have in their micro-contexts of development—such as family, schools, jobs, and neighborhoods—affect their developing civic attitudes (e.g., [25]) and behaviors (e.g., [26]). Young people’s social positions based on their racial and socioeconomic backgrounds interact with their developmental contexts to shape exposure to civic opportunities and civic interests [27]. Contextual investigations often focus on school factors (e.g., “democratic classroom climate”, [28,29]) and community factors (e.g., interactions with the police, civic opportunities, [16,30,31]). Extensive research has shown how civic opportunities are unequally available to young people, especially across racial and socioeconomic backgrounds, in the micro-contexts of schools and communities (e.g., [32,33]), which result from, and perpetuate, disparities in civic preparedness and power.

## 3. Sociopolitical Development among Children from Latinx Immigrant Families

For young people from immigrant families, sociopolitical development has unique elements stemming from being a newcomer, or the children of newcomers, to the United States (US). The legal, social, and political backdrop of immigration in the US is complex, varying across time, country of origin, familial and historical factors, and local and national policies (e.g., [34–37]). Research among young people from Latinx immigrant families has specified how they develop civic attitudes and become engaged in civic behaviors [23,25,38–41]. First-generation immigrants may be positive, even idealistic, regarding their views of the US, and such positive views may decrease with time spent in the US and over generations (e.g., [42]). When people feel unwelcome and excluded from society, as is often the case for young people from Latinx backgrounds in the US, they may develop less positive or more critical attitudes and attachments [43–46]. The congruence or disjuncture between American ideals and one’s own experiences in society predict civic attitude formation (e.g., [31,47]) and cultural identities, which are intersectional and heterogenous, and often motivate civic behavior [23,48,49].

Young people with marginalized identities are often presented with fewer civic opportunities. Specifically, many attend schools with lower access to quality civic education [32], face life circumstances that make extracurricular involvement difficult or impossible (e.g., barriers to transportation, need to participate in paid work, [50]), desire to avoid activities that highlight their ethnic and immigrant status (e.g., [51]), and wish to minimize interactions with immigration and local government officials. However, experiences with marginalization also motivate civic behavior. Social activism can be a coping response to discriminatory experiences [23,39,51]; the “counter-push” hypothesis suggests that many immigrants engage in political and civic activities partly in response to discrimination and experiences with injustice [52,53]. Immigrant youth and the children of immigrants have similar levels of civic motivation and involvement compared to youth from other ethnic and non-immigrant backgrounds [27,39], although many scholars note a need to attend more closely to the diversity within Latinx and other immigrant groups for a more nuanced picture of sociopolitical development (e.g., [24]). Civic attitudes and behaviors in developmental micro-contexts have been explored among youth of color, including those from immigrant families, as well as Latinx backgrounds, but not in the context of farm work.

## 4. Latinx Child Farmworkers in North Carolina

In the sociopolitical landscape of the US, farmworkers are a vulnerable population. Estimates place the number of hired child farmworkers between 30,000 and 80,000 each year [54,55], though the exact number is unknown. Most are people of color, primarily Latinx, and most are immigrants or the children of immigrants [56]. In North Carolina, the Latinx population is rapidly growing [57] and this population is primarily of Mexican descent [58]. Child farmworkers include both migrant workers (meaning they have a change in residence of at least 75 miles for seasonal farm work) and seasonal workers (meaning they live in the same place all year round and work seasonally in agriculture). Farmworkers lack many labor work protections, due to the exclusion of agriculture from many federal and state regulations (i.e., “agricultural exceptionalism”, [59,60]). Many farmworkers lack access to social services resulting from issues of legal authorization (e.g., [61]). Unlike the situation in every other industry in the US, children 12 years old and younger can be hired to perform farm work [62]. Youth farmworkers are exposed to environmental, chemical, physical, and social hazards [63] and experience high rates of work injury and fatality [55,64–66]. Some children hired to work on farms migrate unaccompanied by a parent or legal guardian, lack worker protections such as safety training, and face reduced access to high quality education [50,63,67].

## 5. Farm Work as a Unique Developmental Context for Sociopolitical Development

In addition to the vulnerabilities described, farmworkers face further vulnerabilities that may exclude them from civic life and challenge their sociopolitical development. For example, children often come from families earning low wages, face discrimination, experience language barriers, participate in educational systems not set up for their needs, and have limited access to and time for extracurricular activities [50,68]. Barriers related to immigration status can prohibit some from accessing services [24,69–73]. A national survey of agricultural workers estimated that nearly half of farmworkers lack legal work authorization [74], suggesting that many children hired to do farm work may come from mixed status families. Mixed-status families, where some family members do not have legal documentation while others do, deter young people from civic participation that involves interacting with authorities. Farm work occurs primarily in rural areas, which may further limit civic opportunities relative to urban settings, where youth-serving organizations may be more numerous.

Farmworker social movements in the US illustrate both the structural inequalities that these populations face and their resilience and collective agency to create change and promote the well-being of their communities [61,75,76]. Indeed, farmworkers have many assets that might facilitate sociopolitical development, such as strong social networks within farmworker communities, the potential social and cognitive advantages of bilingualism [77,78], and lived experiences that can motivate collective civic participation [23,40,61,73,79]. Farm work is often conducted in an intergenerational setting [64]. This presents opportunities for youth–adult partnerships [80], the discussion and co-development of sociopolitical understanding [81,82], and passing along historical and social knowledge. However, the hierarchical organization of farm work, involving adult co-workers, crew leaders, and growers, creates a situation for youth where they experience relatively high demands, low control, and low support [83]. Work organized in this manner is set up so that youth are expected to follow rules rather than having a voice in the activities of their daily lives [84,85]. From a developmental perspective, hired farm work is a unique context for development in general and sociopolitical development specifically. Given that agriculture is one of the most hazardous industries in the US, the sociopolitical development of youth workers holds promise for shaping their future advocacy in favor of safe work practices and stronger protections. Yet, Latinx child farmworkers have been an invisible population [86] in most existing sociopolitical development research.

## 6. The Present Study

We examined the civic attitudes and behaviors of hired Latinx children farmworkers in North Carolina. The goals of the present study were to (1) describe civic attitudes and behaviors across relevant demographic characteristics and (2) explore how civic attitudes and behaviors are related in this understudied population and discuss the implications for future research on sociopolitical development among Latinx child farmworkers. Due to the limited prior research on sociopolitical development in this population, we did not specify hypotheses; rather, we intended to document civic attitudes and beliefs among an understudied sample with unique experiences in society.

## 7. Method

### 7.1. Study Design

The data for the present study came from a larger project, the Hired Child Farmworker Study, a community-based participatory research (CBPR) study examining the effects of farm work on the health and development of Latinx child farmworkers in North Carolina. Collaborators include investigators at Student Action with Farmworkers (SAF). With approval from the Wake Forest University School of Medicine Institutional Review Board, participants were recruited in 2017, with follow-up interviews in 2018 and 2019. See [63] for details of the overall CBPR project design, participant recruitment, and data collection procedures. The questions used in the present analysis were asked in the clinic visit portion of the overall study; participants received $40 for the first visit and $50 for the second visit.

### 7.2. Dataset and Analytic Sample

To participate in the *Hired Child Farmworker Study,* participants had to fulfil the following criteria: (1) to be between 10 and 17 years old at the time of the baseline data collection;(2) to self-identify as Latinx; (3) to have worked in farm work in the prior three months (there was no criterion for numbers of hours worked on a farm and participants could still be enrolled in school while participating in farm work); and (4) to be fluent in Spanish or English. The items were translated into Spanish and then back-translated into English to ensure accuracy. The project’s youth advisory board reviewed and pilot tested the surveys to ensure relevance.

No registry of youth farmworkers or other youth farmworker sampling frame exists, making a random sampling approach extremely difficult. Therefore, we used a communityengaged sample design in which community organizations and field interviewers across different North Carolina regions facilitated the recruitment of study participants. As potential participants were identified, the project staff explained the project to parents and children before obtaining signed parental consent and child assent. A small number of the potential participants were “unaccompanied minors”, individuals under the age of 18 years who do not live with a parent or legal guardian [67,87]. The Institutional Review Board approved recruiting “unaccompanied minors” without parental permission.

In 2017, 202 child farmworkers located in 20 North Carolina counties participated in the baseline data collection. Of those, 156 participated in a follow-up data collection survey in 2018 containing the civic attitudes and behaviors measures; an additional 13 completed the survey in 2019. In this manuscript, all the follow-up surveys (whether completed in 2018 and 2019) are referred to as “Time 2”. The analytic sample for this study includes 169 individuals (83.7% of the original 202 participants).

### 7.3. Measures

Demographics. The demographic characteristics include the following: gender (girl,boy), age (continuous and in the categories of 11–13 years, 14–16 years, and 17 years and older); language preferred when interviewed (English or Spanish), country of origin (born in the US or not), and educational attainment (in the categories of 5–7th grade, 8–9th grade, and 10–12th grade). Farm work status was a dichotomous variable (migrant vs. seasonal). Years working in farm work were examined (continuous and in the categories of 1, 2, 3, or 4 or more) along with whether or not the child worked to provide money to their family. Age, educational attainment, and years in farm work were collected at Time 2; all other demographic variables were collected at baseline.

Civic attitudes. Two types of civic attitudes were measured at Time 2. All the items were answered on a scale from 1 (strongly disagree) to 5 (strongly agree). Fair society beliefs were measured through six items: three from Flanagan et al. [28], such as “America is a fair society where everyone has an equal chance to get ahead”, and three from Syvertsen et al. [88], such as “In America, political leaders only listen to the opinions of certain groups” (α = 0.75). The items were reversed so that a high score indicated endorsement of the notion that society is fair (i.e., low awareness of societal inequality). Community connections and efficacy were measured through five items (α = 0.72): three from Syvertsen et al. [88], such as “I can make a positive difference in my community”, and two from Ballard, Cohen & Duarte [89], such as “I contribute to my community”. Participants’ summary scores for fair society beliefs and community connections were constructed by averaging their responses for the items within each measure. These measures have been used with diverse samples including adolescents from Latinx backgrounds [88–90]; they have not been validated for use with child farmworkers.

Civic behaviors. Two types of civic behaviors were measured. Volunteering was measured through three items from the Youth Inventory of Involvement asking participants to rate how often they had participated in activities in the past year, such as “Volunteered with a community service organization” [91,92]. The response options for participation ranged from 1 (rarely) to 5 (very often), and a summary score was calculated for each participant by averaging their responses to the three items. Political activity was measured through nine items from the Youth Inventory of Involvement asking participants to rate how often (rarely to very often) they had participated in activities in the past year. The items included “Expressed my own opinions or beliefs about issues through clothing, buttons or bumper-stickers” and “Attended a protest march, meeting or demonstration”, as well as “Contacted a political representative to tell him/her how you felt about a particular issue” [91,92]. A dichotomous yes/no summary measure for political activity was created for each participant, where political activity was defined as having a positive response of “sometimes”, “often”, or “very often” to at least one of the nine items, while all nine responses equal to “never” or “rarely” was categorized as not engaging in political activity. These items were adapted from previous studies and are intended to measure a wider array of “non-traditional” civic behaviors available to young people in different contexts [91]. They have been used with youth of similar ages and diverse with regards to race/ethnicity and socioeconomic status (e.g., [92,93]); they have not been used previously with child farmworkers.

### 7.4. Statistical Analysis

Descriptive statistics (count, percent or mean, standard deviation as appropriate) were calculated for the demographic variables of interest. For three of the civic variables (fair society beliefs, community connections, and volunteering) means, standard deviations, minimums, and maximums were calculated for summary scores and for the individual items composing those summary scores; agreement was assessed using coefficient alphas. Counts and percentages were used to describe the items composing the political activity civic summary variable. Associations between the demographic variables of interest and the four civic summary variables were calculated using Generalized Linear Models, the Kruskal–Wallis test, or Chi-Square tests, as appropriate. The relations between fair society beliefs, community connections and volunteering, and political activity were examined using correlations or t-tests, as appropriate. With a Bonferroni correction, *p*-values of less than 0.0016 were considered statistically significant.

## 8. Results

Participants in the present study (*n* = 169) were between the ages of 11–19 and 62.7% were boys (see Table 1 for demographic information and Supplemental Table S1 for attribution analysis). Most participants (84.0%) were seasonal farmworkers and 16% were migrant farmworkers; 82% of the participants had participated in farm work for 2 or more years, with the mean being 3.1 years. Most participants (82.8%) reported the US as their country of origin (they were not asked where their parents were born) and the majority of those not born in the US reported being born in Mexico. The majority (87.6%) preferred to complete the interview in English, and a majority (60.4%) reported that they were primarily involved in farm work to contribute to their families.

The participants endorsed a relatively high level of fair society beliefs (M = 2.7, SD = 0.7) and community connections and efficacy (M = 3.6, SD = 0.5) (Table 2). The participants reported a low level of involvement in both volunteering (Median = 2.3, 25th percentile—75th percentile = 1.7–3.0; skew = 0.58 kurtosis =−0.26) and political activity (55% never/rarely and 45% sometimes/often/very often) (Table 2; Supplemental Figure S1).

*Fair society beliefs* were the only civic attitude or behavior that differed across demographic characteristics. Younger participants and those in lower grades reported higher fair society beliefs compared to older participants (Table 3), although this association did not remain significant once adjusted for multiple comparisons.

Volunteering was positively correlated with community connections and efficacy (r = 0.36, *p* < 0.001) and those who reported more involvement in political activity also reported higher feelings of community connections and efficacy (t = −3.1, *p* = 0.002); neither civic behavior was related to fair society beliefs (Table 4).

## 9. Discussion

Describing civic attitudes and behaviors among Latinx children involved in farm work is an important starting point for understanding sociopolitical developmental processes in this context, which is unique in many ways regarding how children shape their views about society. Documenting aspects of sociopolitical development can both highlight the opportunities and needs for these particular children, as well as contribute to broader developmental theory.

The overall pattern of findings for civic attitudes and behaviors suggests that civic *attitudes* are relatively positive and civic *behaviors* are relatively infrequent. On average, the Latinx child farmworkers in this sample endorsed feelings of connection to their community and efficacy within their community more highly, compared to studies with young people from Latinx immigrant backgrounds not involved in farm work [90,94]. These previous studies measured only the sense of efficacy and the current study added items about connections to the community; we infer that this sample of children involved in farm work feel highly connected to their farmworker communities, which might explain this more positive finding. On average, Latinx child farmworkers in this sample endorsed the attitude that society is fair; however, this attitude was lower compared to *connections and efficacy*, indicating some awareness of societal inequality. This attitude was very similar to that found with a similar measure in samples of young people from Latinx immigrant backgrounds not involved in farm work [90,94,95]. Although this relatively positive attitude may reflect experiences of fairness among this sample, in the context of sociopolitical development theory and the realities faced by many farmworkers in the US [61,63], this finding could also reflect motivation to see one’s circumstances as fair, or a lack of opportunity to critically examine attitudes about society. Indeed, aligned with previous research and developmental theory, the only difference in civic attitudes that emerged across the demographic characteristics was that *fair society beliefs* were highest among younger participants. Although these data cannot specify whether this was a cohort effect (owing to these particular groups of youth) or a developmental effect (owing to increasing age), it is plausible that young people develop more critical attitudes about society as they age [96]. This may be especially true for young people who are historically marginalized; as young people age and accumulate more experiences, they may experience or witness more oppression and discrimination [97]. In addition, their cognitive development may facilitate a more critical understanding of systems of inequality. Future longitudinal research can unpack this attitude formation process of sociopolitical development among Latinx youth in the context of farm work.

In terms of behavioral involvement, this sample participated more in volunteering compared to political activities. This aligns with previous studies with middle- and high-school-aged youth, including Latinx immigrants and the children of immigrants [90,94,98]. Involvement in political activities such as contacting representatives was especially low in this sample, with fewer than 10 people reporting such involvement. Young people were more involved in signing petitions and expressing opinions through art or the media; however, participation in these activities was low relative to other studies of slightly older youth, including those from Latinx immigrant backgrounds (e.g., [90]). Prior studies with young immigrants from Latinx backgrounds have found that exposure to injustice may motivate civic action [27,99]; it is possible that the present study sample participants have not yet experienced injustice that motivates civic action, which is consistent with the findings of belief that society is relatively fair (although much lower than connections and efficacy) and low civic action.

Compared to previous studies, the current sample may be different in ways that affect civic behaviors; for example, they are slightly younger and more rural. Children involved in farm work might lack culturally relevant opportunities for such participation, or have competing obligations as they balance school and farm work. They might shy away from political participation that increases contact with immigration or government officials potentially bringing unwanted attention to their families and communities, which may include undocumented individuals. They might be explicitly discouraged from political activities by adults, given fears that political involvement may alienate their employers and given the potential costs of political participation in “hostile” contexts such as conservative areas where anti-immigrant sentiment and tensions with law enforcement are high [100]. The behavioral measures used in the present study may have limited relevance and utility for children involved in farm work. Low involvement in political participation fits with the pattern of civic engagement among youth this age across many demographic groups. Future research could track Latinx child farmworkers over time to understand whether they follow age-related trends of increasing political involvement across late adolescence and young adulthood (e.g., [9]).

In terms of the association between civic attitudes and civic behaviors, the belief that society is fair was not related to either volunteering or political activity, while a sense of connection and efficacy were related to increased instances of both types of civic activity. While past research on sociopolitical development would suggest that awareness of societal inequalities (or low belief that society is fair) can motivate political action [12], this association may not be showing up yet for this young sample, or perhaps this sample experiences too few opportunities for political participation. For this sample of young people working on farms, those who feel more connected and efficacious may be more motivated toward civic participation or may have more opportunities for civic action by virtue of being embedded in communities of farmworkers or other community organizations. Indeed, this sample was partially recruited through a partner organization, which may account for both feelings of social connection and opportunities for civic action. Alternatively, those who are more highly involved in civic behaviors may derive feelings of connection and efficacy from this involvement. Future longitudinal research can disentangle this.

### Limitations and Constraints on Generality

The findings should be considered in the light of the study’s limitations. The measures were chosen based on their previous use with Latinx youth and immigrant populations; however, they have not been normed with farmworkers. Some civic activities may be irrelevant, and others may remain unmeasured. More culturally relevant measures of civic engagement, especially those focused on farmworker populations, are needed. The quantitative and descriptive nature of this analysis presents a starting point; future studies can expand the measures and methods to capture nuance about the context of farm work for civic development. Qualitative data will be particularly useful to highlight the underlying mechanisms. This study examines civic attitudes and behaviors within a specific population; future studies can use comparative strategies to understand how civic development functions in unique and universal ways for Latinx child farmworkers and other youth populations. A great strength of this study is providing data on a very hard-to-reach population; however, generalizability is limited given the specific sample and the non-probability sampling strategy utilized in order to reach the sample. Community organizations facilitated recruitment, and thus the youth in this study may be better connected to resources than youth farmworkers not connected to such organizations. Finally, the study was conducted in one state with a relatively new Latinx population [101] and a specific social context for Latinx child farmworkers [53]. We do not have extensive information on factors such as family country of origin, which may also influence the sociopolitical development of children who are hired to work on farms.

## 10. Implications and Future Directions

The sociopolitical development of historically marginalized populations is important in light of the inequities often experienced [7] across multiple domains including health. Ensuring that youth develop the attitudes, behaviors, and skills to advocate for equity and change, and have the opportunities to do so, is critical, although it is also important to note that the responsibility for changing unequal systems should not rest solely or primarily on marginalized groups but requires broad societal investment. Among the first to examine sociopolitical development in the unique and important developmental micro-context of farm work, this study contributes new knowledge to understanding civic engagement among an often “invisible” population and also extends sociopolitical developmental theory via examination of an understudied micro-context for youth development. Overall, in this sample of Latinx child farmworkers, civic attitudes were relatively positive, including feelings of connection and efficacy, as well as low awareness of inequality, and civic behaviors, including both volunteering and political activities, were relatively infrequent. These findings may imply that the child farmworkers in this sample are developing civic attitudes not yet coupled with civic behavior due to a lack of structural civic opportunities relevant and accessible for them to pursue action. This interpretation makes sense in light of recent findings showing that youth of color, including those from Latinx backgrounds, experience growth in awareness of social inequalities across adolescence [95] and that awareness of social inequalities predicts subsequent civic behaviors, specifically political action, over time [12]. Alternatively, the findings may imply that the study measures are not capturing the relevant civic attitudes and behaviors for samples of youth who work on farms. Future work that is longitudinal, utilizing a more expansive set of measures specifically developed with child farmworkers, will be helpful. It would be especially useful to know what communities these youth are connected to; perhaps farm work communities function as a model for larger community/society, shape attachments, and provide pathways to action in broader community, or, alternatively, working on farms may replace or hinder the formation of attachments and the taking of action in the broader community.

Future work should examine how the daily lives and experiences of child farmworkers inform their developing ideas about civic life in the US and their behavioral participation as they mature. Future studies should also explicate how civic attitudes and behaviors relate to healthy development among children involved in farm work and whether and how sociopolitical development can provide critical tools for advocating for health equity among this population.

## Supplementary Material

Supplemental file

## Figures and Tables

**Table 1.** Descriptive information for survey sample (*n* = 169).

*Children’s Characteristics*	n (%)	Time Point of Measure
Gender		Baseline
Girl	63 (37.3)	
Boy	106 (62.7)	
Age		Time 2
11–13	21 (12.4)	
14–16	78 (46.2)	
17–19	70 (41.4)	
Mean (SD)	15.8 (1.9)	
Primary language		Baseline
English	148 (87.6)	
Spanish	21 (12.4)	
Farm work status		Baseline
Migrant	27 (16.0)	
Seasonal	142 (84.0)	
Country of origin		Baseline
US	140 (82.8)	
Mexico	23 (13.6)	
Guatemala and Honduras	6 (3.6)	
Years in farm work		Time 2
1	30 (17.8)	
2	46 (27.2)	
3	35 (20.7)	
4+	58 (34.3)	
Mean (SD)	3.1 (1.9)	
Work primarily to contribute to family		Baseline
Yes	102 (60.4)	
No	67 (39.6)	
Educational attainment		Time 2
5–7	27 (16.0)	
8–9	50 (29.6)	
10–12	92 (54.4)	

**Table 2.** Descriptive statistics for civic variables (*n* = 169).

	Mean	SD	Min	Max	α
Fair Society Beliefs	2.7	0.7	1.0	4.0	0.75
Fair treatment	2.8	1.1			
Equal chance	3.0	1.2			
Fair society	3.1	1.1			
Not equal chance to participate in government ^1^	2.4	0.9			
Political leaders only listen to certain groups^1^	2.5	0.9			
Certain groups have fewer chances^1^	2.3	0.8			
Community Connections and Efficacy	3.6	0.5	1.6	5.0	0.72
Positive difference	3.8	0.7			
Ways to get involved	4.0	0.7			
Real-life problem solving	3.6	0.8			
Contribute to community	3.4	0.9			
Meaningful role	3.1	0.9			
Volunteering	2.3^2^	1.7–3.0 ^2^	1.0	5.0	0.76
School event	3.0	1.0–4.0			
Community service organization	2.0	1.0–3.0			
Fundraising	2.0	1.0–3.0			
	Never/Rarely		Sometimes/Often/Very Often		
	*n*	%	*n*	%	Mean (SD)
Political Activity	93	55.0	76	45.0	1.4 (0.5)
Signed petition	142	84.0	27	16.0	1.5 (0.9)
Attended a protest march	148	87.6	21	12.4	1.4 (0.8)
Decision to spend money based on a company’s political views	150	88.8	19	11.2	1.3 (0.8)
Expressed opinion through apparel	144	85.2	25	14.8	1.5 (0.9)
Used art or media to express views	141	83.4	28	16.6	1.5 (0.9)
Discussed issues over internet	148	87.6	21	12.4	1.4 (0.8)
Wrote letter	152	89.9	17	10.1	1.3 (0.7)
Contacted representative	161	95.3	8	4.7	1.1 (0.5)
Campaigned	164	97.0	5	3.0	1.1 (0.5)

1 Reverse coded.

2 Median/25% percentile–75% percentile.

**Table 3.** Differences in civic variables across demographic groups (*n* = 169).

	Fair Society Beliefs		Community Connections and Efficacy		Volunteering		Political Activity^1^	
Gender	Mean	SD	Mean	SD	Median	P25-P75	*n*	%
Girl	2.6	0.8	3.6	0.5	2.3	1.7–3.7	28	44.4
Boy	2.7	0.6	3.6	0.5	2.3	1.7–3.0	48	45.3
Age								
11–13	3.0 ^a^	0.6	3.7	0.5	2.3	1.3–2.7	11	52.4
14–16	2.7^a,b^	0.7	3.5	0.6	2.3	1.7–3.0	30	38.5
17–19	2.5^b^	0.7	3.6	0.5	2.3	1.7–3.0	35	50.0
Primary language								
English	2.7	0.7	3.6	0.5	2.3	1.7–3.0	66	44.6
Spanish	2.7	0.7	3.6	0.6	2.3	1.7–3.0	10	47.6
Farm work status								
Migrant	2.9 ^a^	0.6	3.5	0.6	2.0	1.7–2.7	12	44.4
Seasonal	2.6	0.7	3.6	0.5	2.3	1.7–3.0	64	45.1
Country of origin								
US	2.7	0.7	3.6	0.5	2.3	1.7–3.0	63	45.0
* Other	2.5	0.8	3.6	0.6	2.3	1.7–3.0	13	44.8
Years in farm work								
1	2.5	0.8	3.7	0.5	2.7	1.7–4.0	13	43.3
2	2.6	0.6	3.6	0.5	2.2	1.7–3.0	24	52.2
3	2.8	0.6	3.5	0.6	2.3	1.7–3.0	15	42.9
4+	2.8	0.7	3.6	0.6	2.3	1.7–3.0	24	41.4
Work primarily to contribute to family								
Yes	2.7	0.7	3.6	0.6	2.3	1.7–3.0	47	46.1
No	2.6	0.7	3.6	0.5	2.3	1.7–3.0	29	43.3
Grade in School								
5–7	3.1 ^a^	0.6	3.6	0.6	2.0	1.7–2.7	14	51.9
8–9	2.6 ^b^	0.7	3.6	0.6	2.3	1.7–3.0	18	36.0
10–12	2.6 ^b^	0.7	3.6	0.5	2.3	1.7–3.0	44	47.8

*Notes*. Groups with different alphabetic superscripts within a column different significantly from each other.

* For this analysis, participants born in Mexico, Honduras, and Guatemala were grouped together in the category “other country of origin” due to small sample size. *p* < 0.01; no significant differences were detected for with a Bonferroni correction of *p* < 0.0016.

1 Sometimes/often/very often (*n* = 76).

**Table 4.** Correlations (or t-tests) between civic attitudes and behaviors.

	Fair Society Beliefs		Community Connections and Efficacy	
Volunteering	r	*p*-value	r	*p*-value
	−0.008	0.91	0.36	<0.0001
Political activity	Mean (SD)		Mean (SD)	
Sometimes/Often/Very Often	2.6 (0.7)	t = 0.62	3.7 (0.5)	t = −3.12
Never/Rarely	2.7 (0.7)	(*p* = 0.53)	3.5 (0.6)	(*p* = 0.002)

## Data Availability

Datasets presented in this article are not readily available due to technical/time concerns. Request to access the datasets should be directed to Dr. Stephanie Daniel (sdaniel@wakehealth.edu).
