# Supplementary material for: Sociopolitical Development among Latinx Child Farmworkers
Source: Youth (Basel). Author manuscript; Available in PMC 2025 May 29. (PMC12121946; doi:10.3390/youth4020037)

**Supplemental Material.**

**Supplemental Table 1.** Descriptive information of study sample at baseline n(%)

| *Children’s Characteristics* | Full sample  (n = 202) | Civic sample  (n = 169) | Baseline only (n = 33) | p-value |
| --- | --- | --- | --- | --- |
| Gender |  |  |  | 0.8185 |
| Girl | 76 (37.6) | 63 (37.3) | 13 (39.4) |  |
| Boy | 126 (62.4) | 106 (62.7) | 20 (60.6) |  |
| Age  11-13  14-16  17-19  Mean (SD) | 45 (22.3)  110 (54.4)  47 (23.3)  14.9 (1.8) | 41 (24.3)  93 (55.0)  35 (20.7)  14.8 (1.8) | 4 (12.1)  17 (51.5)  12 (36.4)  15.6 (1.8) | 0.0909 |
| Primary language |  |  |  | 0.0564 |
| English | 172 (85.2) | 148 (87.6) | 24 (72.7) |  |
| Spanish | 30 (14.8) | 21 (12.4) | 9 (27.3) |  |
| Farm work status |  |  |  | 0.1209 |
| Migrant | 36 (17.8) | 27 (16.0) | 9 (27.3) |  |
| Seasonal | 166 (82.2) | 142 (84.0) | 24 (72.7) |  |
| Country of origin |  |  |  | 0.1739^1^ |
| US | 164 (81.2) | 140 (82.8) | 24 (72.7) |  |
| Mexico | 26 (12.9) | 23 (13.6) | 3 (9.1) |  |
| Guatemala/Honduras/El Salvador | 12 (5.9) | 6 (3.6) | 6 (18.2) |  |
| Years in farm work  1  2  3  4+  Mean (SD) | 70 (34.7)  53 (26.2)  42 (20.8)  37 (18.3)  2.5 (1.7) | 61 (36.1)  41 (24.3)  37 (21.9)  30 (17.7)  2.5 (1.8) | 9 (27.3)  12 (36.4)  5 (15.2)  7 (21.2)  2.5 (1.4) | 0.4001 |
| Work primarily to contribute to family |  |  |  | 0.9785 |
| Yes | 122 (60.4) | 102 (60.4) | 20 (60.6) |  |
| No | 80 (39.6) | 67 (39.6) | 13 (39.4) |  |
| Educational attainment |  |  |  | 0.1428 |
| 3-7  8-9  10-12 | 43 (21.3)  74 (36.6)  85 (42.1) | 38 (22.5)  65 (38.5)  66 (39.0) | 5 (15.1)  9 (27.3)  19 (57.6) |  |

^1^US vs. Non-US

**Supplemental Figure 1.** Detail on Volunteering: Frequencies and Skew


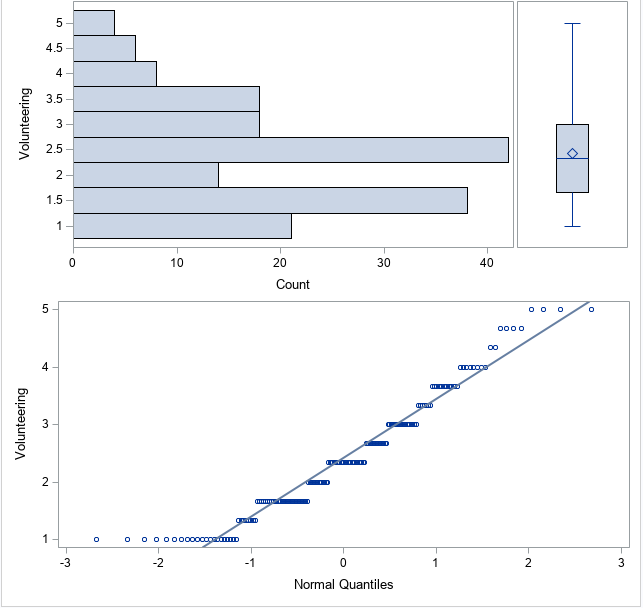

Supplement: Supplemental file [file NIHMS2040650-supplement-Supplemental_file.docx]
